# Dissecting schizophrenia phenotypic variation: the contribution of genetic variation, environmental exposures, and gene–environment interactions

**DOI:** 10.1038/s41537-022-00257-5

**Published:** 2022-05-10

**Authors:** Hanxin Zhang, Atif Khan, Steven A. Kushner, Andrey Rzhetsky

**Affiliations:** 1 Committee on Genetics, Genomics and Systems Biology, The University of Chicago, Chicago, IL 60637 USA; 2 Department of Medicine, and Institute of Genomics and Systems Biology, The University of Chicago, Chicago, IL 60637 USA; 3 Department of Psychiatry, Columbia University, New York, NY USA; 4 New York State Psychiatric Institute, New York, NY USA; 5 Department of Psychiatry, Erasmus MC, University Medical Center Rotterdam, Rotterdam, the Netherlands; 6 Department of Human Genetics and Committee on Quantitative Methods in Social, Behavioral, and Health Sciences, The University of Chicago, Chicago, IL 60637 USA

**Keywords:** Genetics of the nervous system, Schizophrenia

## Abstract

Schizophrenia is among the leading causes of disability worldwide. Prior studies have conclusively demonstrated that the etiology of schizophrenia contains a strong genetic component. However, the understanding of environmental contributions and gene–environment interactions have remained less well understood. Here, we estimated the genetic and environmental contributions to schizophrenia risk using a unique combination of data sources and mathematical models. We used the administrative health records of 481,657 U.S. individuals organized into 128,989 families. In addition, we employed rich geographically specific measures of air, water, and land quality across the United States. Using models of progressively increasing complexity, we examined both linear and non-linear contributions of genetic variation and environmental exposures to schizophrenia risk. Our results demonstrate that heritability estimates differ significantly when gene–environment interactions are included in the models, dropping from 79% for the simplest model, to 46% in the best-fit model which included the full set of linear and non-linear parameters. Taken together, these findings suggest that environmental factors are an important source of explanatory variance underlying schizophrenia risk. Future studies are warranted to further explore linear and non-linear environmental contributions to schizophrenia risk and investigate the causality of these associations.

## Introduction

Schizophrenia is a chronic, debilitating psychiatric disorder with high heritability and among the leading causes of disability worldwide^1^. While knowledge of genetic determinants of schizophrenia risk has revealed novel insight into the underlying disease biology, elucidation of environmental determinants has the potential to not only contribute to our etiologic and pathophysiological understanding, but perhaps more compellingly, to address potential modifiable environmental risk factors that might be useful in primary, secondary, and tertiary prevention.

Schizophrenia heritability has been estimated using classical twin studies as greater than 70%^2,3^, while more recent epidemiological birth registry studies have provided estimates of about 60%^4^. Considered together with an observed monozygotic twin concordance of ~50%, environmental factors and gene–environment interactions have been hypothesized to explain a considerable amount of the variance underlying schizophrenia risk. However, few studies of schizophrenia have attempted to directly model the relative contributions of genetics, environment, and gene–environment interactions. Among the major hurdles for directly estimating the contribution of environmental effects on schizophrenia risk has been the limited availability of high-quality quantitative data of spatially varying environmental variables standardized across a sufficiently large geographical region.

It is broadly understood that the accuracy of estimates of the relative contribution of genetic variation, environmental exposures, and gene–environment interactions to phenotypic variation strongly depends on the richness of the underlying data and the accuracy of the modeling assumptions. In particular, the operational definition of “environment” ranges from quantified endpoints in the physical environment to anything other than genes (see Supplemental Materials, Environmental Factors in Schizophrenia). From a clinical perspective, it would be useful to distinguish pure heritability from interactions between genetic variation and environmental stimuli, and ideally, to identify environmental triggers that contribute to disease onset and progression in predisposed individuals. Here, we use data from nearly half a million US individuals, organized into nuclear families to estimate schizophrenia liability using a progression of increasingly complex models of disease inheritance, taking into account explicit environmental^5,6^, geographic, and socioeconomic data. Our findings suggest that a substantial proportion of the variance underlying schizophrenia risk is explained by interactions between genetic variation and environmental factors.

## Methods

### Data

We used the IBM Health MarketScan dataset^7^, which records the health histories of nearly half of the US population from 2003 to 2018. The adult portion of the MarketScan cohort includes records for enrollees over 16 years old, which covers from the beginning of 2003 and to the end of 2018 (data release in 2019). This dataset provides health records for 144,989,035 adults (at least 16 years old), 276,856 of which have at least one code of schizophrenia-related diagnoses (0.19%), and 177,937 individuals possess two or more diagnostic codes (0.12%).

The data documents both nuclear family structure and places of residence for enrollees (US county level), allowing us to infer genetic relationships and environmental quality measures associated with geographic locations. We selected individuals who consistently lived in the same location, and therefore were exposed to the same geographical environment, for at least six years. After this selection process, there remained 128,989 families (481,657 individuals), including parents and their children older than 16-year old.

We used a collection of county-level environmental quality indices (EQIs) developed by the US Environmental Protection Agency (EPA) to quantify the fixed environmental factors^8,9^. There are five EQI domains: air, water, land, sociodemographic, and human-built environment. Each domain index summarizes relevant variables using a principal component analysis (PCA), for which lower index scores are indicative of generally higher environmental quality (see Supplementary Data 1 for complete list of variables). For the air, water, and land domains, EQIs represent overall quality based on the measurement of a wide diversity of pollutants and contaminants. The sociodemographic domain represents environmental quality related to income, education, employment, crime, and other socioeconomic elements. The built-environment domain summarizes factors related to housing quality, proximity to roads, and the intensity of traffic on these roads.

#### Ethics

The study involved de-identified data and was approved as IRB-exempt by the University of Chicago IRB.

### Modeling

We applied the following logic for estimating proportions of contribution of genetic variation, environmental factors, and their interactions from family pedigrees and phenotypic information. Consider a nuclear family with two children who are full siblings and common offspring of the parents, who are assumed to be unrelated. Each child shares on average half of their respective genetic variation with the nuclear genomes of both parents, as well as with each other. The model considers shared environmental exposures with respect to distinct genetic relationships: the family as whole (environment could vary between families), shared by siblings, shared by parents, and specific to an individual. In addition, the model also considers the socioeconomic and demographic surroundings of each family. By examining each combinatorial category regarding disease status of each family, the model partitions overall phenotypic variance into portions attributable to genetics (heritability), to components of environment, and to their interactions. For a nuclear family of *n* people, there are *n!* possible familial disease status configurations. We utilized generalized linear models (GLMs), in which partitioning of variance is accomplished with random effect variables, while measured demographic, socioeconomic, and environmental confounders are represented by fixed effect parameters.

We designed generalized linear mixed-effects models to infer the ways different factors contribute to the etiology of schizophrenia. The fixed-effects segment included basic demographic factors (sex and age) along with the EQIs split by category: air, water, land, sociodemographic, and built-environmental. We designed the random-effects segment to evaluate how much of the outcome’s variance could be explained by genetic composition, environmental variables, and gene–environment interactions. By forwardly adding terms, we constructed a sequential progression of increasingly complex models. Supplementary Table 1 summarizes the additive effects considered in each model. We categorized the models into two forward selection traces: a) Linear model 0 (LM0) ⟶ Linear model 1 (LM1) ⟶ Interaction model 1 (IM1), and b) Linear model 2 (LM2) ⟶ Interaction model 2 (IM2). Within each trace, the latter models encompass all of the preceding models’ variables. The forward selection traces allowed us to determine whether models with additional complexity could better fit the data by comparing the information criterion estimates.

In Supplementary Table 1, we report the statistics *p*^*2*^*, h*^*2*^*, f*^*2*^*, c*^*2*^*, s*^*2*^*, e*^*2*^*, hf*^*2*^*, hc*^*2*^*, hs*^*2*^, and *he*^*2*^, which represent the relative proportion of outcome variation attributable to geographic residence (*p*^*2*^), genetics (*h*^*2*^), familial environment (*f*^*2*^), couple-shared environment (*c*^*2*^), sibling-shared environment (*s*^*2*^), individually independent environment (*e*^*2*^), interactions between genetics and familial environment (*hf*^*2*^), interactions between genetics and couple-shared environment (*hc*^*2*^), interactions between genetics and sibling-shared environment (*hs*^*2*^), and interactions between genetics and individual environment (*he*^*2*^). The Models section of the Supplementary Materials provides further details regarding model definitions.

## Results

**Fig. 1:**
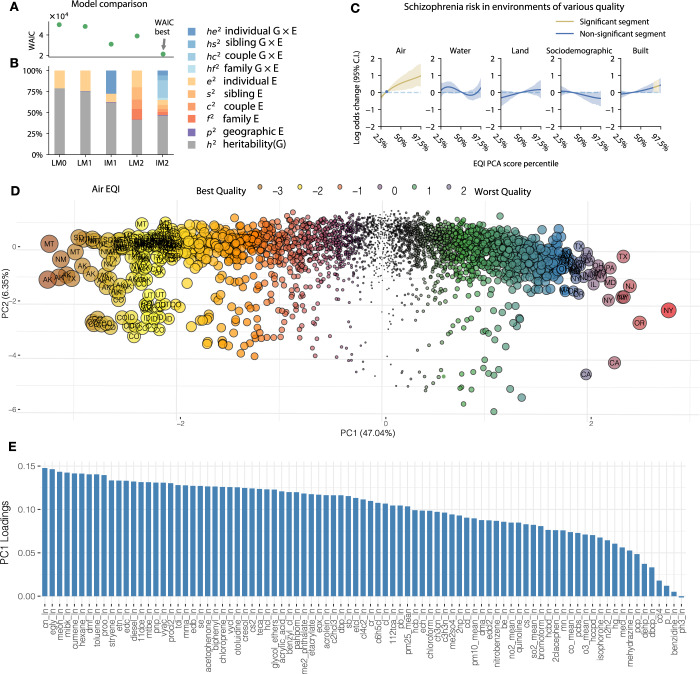
Comparing five models of phenotypic variation of schizophrenia. Comparing model fit across a collection of increasingly complex models of schizophrenia heritability: model specific WAIC values (**a**) are juxtaposed with corresponding heritability estimates (**b**), also provided in Supplementary Table 2. **a** WAIC values quantify goodness-of-fit of the corresponding models, with a lower WAIC associated with a better model fit. **b** Bar plot showing the proportion of outcome variance explained by each effect variable, grouped into four major categories with shaded sub-categories: heritability (gray bars), geographic location (violet bars), environmental factors (yellow-orange bars), and gene–environment interactions (blue bars). **c** Effect of environmental variables on schizophrenia risk. Plot shows the non-linear effects estimated for the five EQI domains (air, water, land, sociodemographic, and built-environment) based on the WAIC-best model, IM2. To visualize the IM2 results, we converted the EQI scores (PC1 generated by principal component analyses) to percentiles based on the population distributions and used the 2.5th to 97.5th percentiles as the *x*-axis limits of the plots. Blue and olive segments indicate significant and non-significant EQI regions that are associated with the log-odds of schizophrenia, respectively. **d** Principal component scores of air quality across US counties, each dot in the figure correspond to a unique US county where the size of the dot is proportional to their distance from the mean. Selected counties in either extremities are labelled with their state abbreviations. The U.S. EPA computed county-level air quality index using scores of PC1 as proxy for air quality. Red, purple, and blue dots represent counties with poorer air quality (higher PC1 scores). Brown, yellow, or orange dots represent counties with higher air quality (lower PC1 scores). Counties with relatively extreme air quality measures are shown with larger dots. **e** Loading of the first principal component summarizing a wide range of air pollutants. The full list of air quality indicators is provided in Supplemental Data 1 along with explanation of each measurement meaning.

We fit models using a Bayesian framework (see Supplementary Materials). Supplementary Table 2 shows the mean estimates of the heritability and environmental statistics for all models. To rank models with respect to their goodness of fit, we used the Widely-Applicable Information Criterion (WAIC) (see Supplementary Materials, Models). Models with the smallest WAIC provide the optimum balance between complexity and explanatory power^10^. Figure 1 shows heritability estimates juxtaposed with model-specific WAIC values, which are also provided in Supplementary Table 2.

The interaction models (IM1 and IM2) provided a superior fit compared with their corresponding linear models (LM1 and LM2) (Fig. 1A). Notably, the most complex interaction model, IM2, had the lowest WAIC estimate of all models examined. Accordingly, gene–environment interactions explained a substantial proportion of the total phenotypic variance in both models that included interaction terms (IM1: 28%, IM2: 35%; Fig. 1B).

Heritability estimates differed significantly between no-interaction (“linear”) and interaction models. The simplest linear model (LM0) yielded the highest heritability estimate (79%). More complex models, and especially models with gene–environment interaction terms, generated lower heritability estimates. The best-fit model, IM2, incorporating all environmental and interaction effects, yielded the lowest heritability (46%).

We also investigated how environmental factors, estimated as fixed effects, influenced the log-odds of schizophrenia. To visualize IM2 results, we converted the EQI scores (PC1 generated by principal component analyses) to percentiles based on the population distributions and used the 2.5th to 97.5th percentiles as the *x*-axis limits of the plots. Figure 1C shows the non-linear effects estimated for the five EQI domains (air, water, land, sociodemographic, and built-environment) based on the WAIC-best model, IM2. We found that a lower quality of air and built environments were associated with a higher risk of schizophrenia. However, we discovered no significant associations of schizophrenia rates with environmental quality of water, land, or sociodemographic factors (Fig. 1c). Figure 1d shows the distribution of principal components 1 and 2 scores (PC1 and PC2) of air quality measurements across US counties, with higher scores of PC1 representing poorer air quality. The principal component loadings (PC1) of the 87 individual pollutants contributing to the air quality index are shown in Fig. 1e (see Supplementary Data 1 for complete details). We conjectured that various pollutants vary in a collinear way, captured by the component of the principal component analysis.

The proportion of variance contributed by geographic residence was consistently small (*p*^*2*^ < 1%) across all four models (Fig. 1). These results suggest that the five EQI domains, modeled in the fixed-effects segment, are likely to account for most of the outcome variation associated with geographical residence, given the strong co-variation with environmental qualities.

## Discussion

Using a Bayesian framework, we implemented a series of increasingly complex models to estimate the relative contributions of genetic factors, environmental factors, and gene–environment interactions to the variance underlying schizophrenia liability. We found that the most complex model (IM2), containing the full repertoire of environmental and gene–environment interaction terms, appeared most optimal given that it had the lowest widely applicable information criterion (WAIC) value. The heritability estimate under IM2 was 46%, with much of the remaining variance explained by the gene–environment interaction terms. Among the five environmental quality indices (EQIs) examined, air, and to a lesser extent built, domains were significantly associated with schizophrenia risk.

The list of previously investigated candidate environmental determinants of schizophrenia risk is extensive and has typically been biased towards childhood and prenatal exposures (see Supplementary Materials, Environmental Factors in Schizophrenia). Almost certainly, there are additional environmental factors and gene–environment interactions yet to be discovered. The association between air pollution and risk of schizophrenia is a topic of emerging consensus. Evidence from recent epidemiological studies have suggested that exposure to air pollution may adversely affect the brain and broadly increase the risk for psychiatric disorders, including schizophrenia^11–13^. Analogous to the ongoing elucidation of the genetic architecture of schizophrenia, in which variants of larger effect were the first to be identified, disproportionate attention has thus far been focused on highly adverse or traumatic events occurring during early life. Our findings provide additional candidate environmental factors that warrant further investigation, in particular with regard to potential causality, given that many of the factors we have studied are in principle modifiable.

Notably, in models that included quantitative EQIs, our estimates of schizophrenia heritability were somewhat lower than prior studies. Specifically, in the IM2 model that included the full repertoire of environmental variables and gene–environment interactions, heritability accounted for only 46% of the variance underlying schizophrenia risk, in contrast to prior studies that generally yielded estimates of 60–85%, while gene–environment interactions explained the majority the remaining variance (35%) unaccounted for by genetics alone and additive environmental effects explained 19% (Supplementary Table 2). Moreover, within the sub-categories we examined of gene–environment interactions, *hc*^*2*^ (interactions between genetics and couple-shared environment) had the largest contribution (21%), which is likely to reflect assortative mating.

Our study has limitations. The analysis relies upon family structure to dissect the relative contribution of genetic and environmental factors, along with their interactions. Ideally, we would have been able to utilize whole genome and mitochondrial sequencing data for everyone in the cohort, in addition to a comprehensive quantitative history of individual-level environmental exposures. Specifically, the available environmental exposures within our dataset lacked records for at least a sizeable proportion of highly salient events that have previously been identified as candidate determinants of schizophrenia risk, including physical and emotional trauma, poverty, or loss of close relatives. Unfortunately, to our knowledge such data is not currently available in any cohorts at the order of magnitude scale we have examined, though considerable focused efforts are being continually made to further enhance the depth, breadth, and precision of the available population-level data. Therefore, we used a Bayesian model to partition phenotypic variance into etiological groups. However, we acknowledge that we cannot establish the causality for any of the identified associations underlying schizophrenia risk.

Future work should be done to investigate higher-order interactions of genetic variants and environmental factors, to expand further the breadth of quantitative environmental metrics, and to assess the causality of the identified environmental associations and gene–environment interactions. Such knowledge would have the potential to implement preventative public health measures at the level of the general population, as well as personalized clinical strategies through genotype-guided primary, secondary and tertiary prevention to protect defined individuals from exposure to specific environmental risks.

## Supplementary information

Supplementary Methods

EPA data

## Data Availability

All results needed to evaluate the conclusions are either reported in the manuscript text, figures, and/or Supplementary Materials. The source code and data used for analysis can be accessed at the project web site: https://github.com/hanxinzhang/schizophrenia-etiology.
