# Supplementary material for: Dissecting schizophrenia phenotypic variation: the contribution of genetic variation, environmental exposures, and gene–environment interactions
Source: Schizophrenia (Heidelb). 2022 May 10;8(1):51. doi: 10.1038/s41537-022-00257-5 (PMC9261082; doi:10.1038/s41537-022-00257-5)
Supplement: Supplementary file 1 — Supplementary Methods [file 41537_2022_257_MOESM1_ESM.docx]

## Supplementary Materials

### Environmental Factors in Schizophrenia

The early-stage (conception to birth) factors believed to contribute to the disorder’s onset include: season of conception ^1,2^, prenatal famine ^3^, rhesus incompatibility of a male fetus with the mother ^4^, obstetrical complications -- especially associated with hypoxia (fetal oxygen deprivation) ^5-9^, and maternal stress during pregnancy ^10-14^ (which also presents in animal models ^15^). Infections also feature prominently as possible triggers, presumably affecting a pregnant mother during the gestation of her fetus ^16^. Among the most likely causal infections are influenza ^17^, rubella ^18^, and poliovirus ^19^. Other early-stage triggers may include a patient’s low birth weight ^20^, preterm birth ^21^, maternal depression ^22^, maternal vitamin D deficiency ^23^, social class at birth ^24^, and environmental pollution. ^25^

There are also childhood environmental factors proposed to contribute to onset of the disorder, including events triggering chronic low-grade inflammation ^26^ and immunity-affecting stressors ^27^, meningitis ^28^, toxoplasmosis ^29^, early-life sexual abuse ^30^, oxidative and nitrosative stress ^31^, physical and psychological traumas ^32^, and stress ^33,34^ such as the absence, death ^35^, or institutionalization of a parent ^36^, and parental maltreatment ^37^.

In an individual’s later life, the tentative environmental factors contributing to schizophrenia onset include use of cannabis ^38^, vitamin B deficiency ^39^, traumatic brain injury ^40^, substance abuse ^41^, urbanicity ^42^, and immigrant or minority status ^43^.

### Models

Linear model 0 (LM0)

For a phenotypic disease, we assumed that the probability of presenting this disease is . The logit-probability, defined as , could be expressed in an additive mixed-effects model:

|  |  | (1) |
| --- | --- | --- |

where is the design matrix of the demographic fixed-effects, including the effects of age and sex. For the fixed effect controlled by environmental quality indices , we assumed a polynomial model:

|  |  | (2) |
| --- | --- | --- |

Our model includes five () different types of environmental qualities quantified by summary indices: air, water, land, sociodemographic, and build-environment domains. We set the degree of the polynomial function and its components … to be three (cubic).

is the genetic effect contributing to the phenotype, and is the individual-specific environmental effect. For an individual group, their genetic effects are associated by the genetic relationship matrix (GRM, ). As an example, the GRM for a family of two parents and one child should be close to

|  |  | (3) |
| --- | --- | --- |

where the first two rows and columns represent the parents, and the last row and column represents the child. Then, we assumed the genetic effects followed a multivariate normal distribution:

|  |  | (4) |
| --- | --- | --- |

The individua environmental effects were also assumed to follow a multivariate normal distribution with their covariances equal to a multiple of the identity matrix :

|  |  | (5) |
| --- | --- | --- |

In these expressions, and are the constants we set out to find. For a population, the variance-covariance of is

|  |  | (6) |
| --- | --- | --- |

Finally, we defined the heritability and the independent environmental factor as

|  |  | (7.1) |
| --- | --- | --- |
|  |  | (7.2) |

Linear model 1 (LM1)

Besides the effects contributed by the basic demographic information (sex and age) and environmental quality, we acknowledged that geographic position might also play a part in disease etiology ^44^.

Given a patient’s dwelling’s latitude and longitude (coordinates) ), the random effect, as a part of the logit-probability , is that follows a Gaussian process (GP):

|  |  | (8) |
| --- | --- | --- |

The Gaussian process model constrains the distribution of , so that the joint distribution of two data points and is a multivariate normal:

|  |  | (9) |
| --- | --- | --- |

Therefore, if we choose the kernel function appropriately, we can fit a function that makes two random effects and correlated according to the proximity of coordinates and . Here, we use the Exponentiated Quadratic kernel function, which is commonly used in geo-statistics requiring a smooth metric space:

|  |  | (10) |
| --- | --- | --- |

where and are the scale parameters, we fit through the Markov chain Monte Carlo (MCMC) process.

In all, as a forward addition to the simplest linear model 0 (LM0), we expressed the logit-probability of presenting a disease for an individual as:

|  |  | (11) |
| --- | --- | --- |

The variance-covariance of for a group of individuals is:

|  |  | (12) |
| --- | --- | --- |

where is the covariance matrix capturing individual variation of phenotype according to the individual’s geographic position. This covariance matrix is derived from the kernel function, Expression (10), as we did in Expression (9).

Consequently, the geographic position factor , which quantifies how much variation could be explained by one’s coordinates, the heritability , and the independent environmental factor are

|  |  | (13.1) |
| --- | --- | --- |
|  |  | (13.2) |
|  |  | (13.3) |

Interaction Model 1 (IM1)

We then considered the interaction between the genetic effect and the environmental effect. The logit-probability of having a disease can be expressed as

|  |  | (14) |
| --- | --- | --- |

The scale factor determines how much effect the interaction contributes to the phenotype. and are the same genetic and environmental effects as they were given in the linear models 0 and 1. The variance-covariance matrix of was defined as follows.

|  |  | (15) |
| --- | --- | --- |

Where we are assuming that the genetic effect and the environmental effect are statistically independent. The operator represents the elementwise (Hadamard) product. The geographic position factor , the heritability , and the independent environmental factor are

|  |  | (16.1) |
| --- | --- | --- |
|  |  | (16.2) |
|  |  | (16.3) |

Furthermore, interactions between genetics and the environment can also explain the variance of the logit-probability. We have defined a new interaction factor

|  |  | (17) |
| --- | --- | --- |

Linear Model 2 (LM2)

The linear models 0 and 1 incorporate the individual environmental effect only. However, because family members, couples, and siblings may have shared similar behaviors and milieus, we also included other types of environmental effects in our model. Here, the linear model 2 considers the family effect , the couple effect , and the sibling effect in addition to the individual environmental effect :

|  |  | (18) |
| --- | --- | --- |

These additional environmental effects were also assumed to follow multivariate normal distributions,

|  |  | (19.1) |
| --- | --- | --- |
|  |  | (19.2) |
|  |  | (19.3) |

We determined the relationship matrices , , and by how much of the kinship-specific environment they shared on average. The relationship matrices for a family of two parents (first two rows and columns) and two children (last two rows and columns) are:

|  |  | (20.1) |
| --- | --- | --- |
|  |  | (20.2) |
|  |  | (20.3) |

We can then estimate the variance-covariance of as:

|  |  | (21) |
| --- | --- | --- |

We defined the heritability and other statistics specifying environmental effects as:

|  |  | (22.1) |
| --- | --- | --- |
|  |  | (22.2) |
|  |  | (22.3) |
|  |  | (22.4) |
|  |  | (22.5) |
|  |  | (22.6) |

Interaction Model 2 (IM2)

Similar to our process in the experimental models 1 and 2, we added interaction terms into the above-defined linear model 2, thus the logit-probability of having a disease is:

|  |  | (23) |
| --- | --- | --- |

The variance-covariance of is:

|  |  | (24) |
| --- | --- | --- |

We defined the heritability and statistics specifying the environmental effects (geographic position: , family: , couple: , sibling: , and independent: ) as we did in Expressions 16 and 22. Likewise, statistics quantifying the genetic interactions (genetics-family: , genetics-couple: , genetics-sibling: , and genetics-individual-environment: ) are given according to their partitions in , similar to the Expression 17.

### Bayesian inference

Priors

To find the posterior distribution of parameter under a Bayesian framework, we first needed to specify the prior according to the Bayes theorem . We used the horseshoe shrinkage prior^45^ for the fixed-effects parameters (like the demographic predictor parameter in the Expression 1 and the polynomial function parameter in Expression 2), so we imposed sparsity and regularization to avoid overly complicated models. For parameters restricted to be positive, such as the variance scale factor and in Expression 6, we used the zero-avoiding Gamma prior, as recommended by Chung et al. ^46^ and the STAN prior choice recommendations ^47^.

Sampling

After specifying the hierarchical models with priors and likelihood functions, Bayesians rely on a sampling algorithm (sampler) to draw from the posterior distribution and approximate the posterior efficiently. In the present study, we used Hoffman and Gelman’s No-U-Turn sampler (NUTS) ^48^, which was designed for high-dimensional, ill-shaped target distributions. The No-U-Turn sampler tunes the step size automatically and directs sampling by looking to the gradient information. Compared to vanilla Markov chain Monte Carlo methods, the No-U-Turn sampler ^48^ depicts complex posterior distributions more quickly. We initialized the sampling process using automatic differentiation variational inference (ADVI) ^49^, providing the MCMC with an optimized start similar to the Frequentist’s maximum likelihood methods ^50^.

### Supplementary figures and tables

**Supplementary Table 1: Model set-ups and statistics**

| **Model** | **Fixed effects** | **Random effects** | **Corresponding statistics** |
| --- | --- | --- | --- |
| **Linear model 0** | Demo + Env | G + E | *h^2^, e^2^* |
| **Linear model 1** | Demo + Env | Geo + G + E | *p^2^, h^2^, e^2^* |
| **Interaction model 1** | Demo + Env | Geo + G + E + GE | *p^2^, h^2^, e^2^, he^2^* |
| **Linear model 2** | Demo + Env | Geo + G + F + C + S + E | *p^2^, h^2^, f^2^, c^2^, s^2^, e^2^* |
| **Interaction model 2** | Demo + Env | Geo + G + F + C + S + E + GF + GC + GS + GE | *p^2^, h^2^, f^2^, c^2^, s^2^, e^2^, hf^2^, hc^2^, hs^2^, he^2^* |
| **The fixed-effect terms are**  Sex + Age (Demo)  Environmental quality indices (Env)  **The random effect terms are defined by the partition of the phenotype explained by:**  Geo: geographic position, described by coordinates (latitude and longitude)  G: genetics  E: the individually-independent environment  F: the environment shared by family members  C: the environment shared by couples  S: the environment shared by siblings  GE: the interaction between genetics and the individually-independent environment  GF: the interaction between genetics and the family-shared environment  GC: the interaction between genetics and the couples-shared environment  GS: the interaction between genetics and the siblings-shared environment | | | |

**Supplementary Table 2: Mean estimates and 95 C.I. of heritability and environmental statistics**

|  | ***p^2^*** | ***h^2^*** | ***f^2^*** | ***c^2^*** | ***s^2^*** | ***e^2^*** | ***hf^2^*** | ***hc^2^*** | ***hs^2^*** | ***he^2^*** | **WAIC** |
| --- | --- | --- | --- | --- | --- | --- | --- | --- | --- | --- | --- |
| **LM0** |  | 79  (64, 93)% |  |  |  | 21  (6.6, 36)% |  |  |  |  | 50015.45 |
| **LM1** | 0.35  (0.072, 0.71)% | 75  (64, 88)% |  |  |  | 24  (13, 36)% |  |  |  |  | 48294.41 |
| **IM1** | 0.89  (0.15, 1.8)% | 61  (39, 84)% |  |  |  | 10  (2.2, 19)% |  |  |  | 28  (0.0, 45)% | 31062.84 |
| **LM2** | 0.31  (0.049, 0.66)% | 41  (29, 59)% | 13  (4.8, 19)% | 11  (5.0, 19)% | 15  (0.94, 27)% | 20  (4.9, 33)% |  |  |  |  | 39021.58 |
| **IM2** | 0.77  (0.18, 1.5)% | 46  (23, 67)% | 3.7  (0.020, 9.3)% | 3.5  (0.036, 11)% | 4.9  (0.034, 13)% | 6.0  (0.68, 13)% | 2.5  (0.0, 8.6)% | 21  (9.4, 31)% | 5.8  (0.0, 21)% | 5.8  (0.0, 18)% | 21434.43 |
| **The statistics are defined by the partition of the phenotype explained by**  ***p^2^*: geographic position, described by coordinates (latitude and longitude)**  ***h^2^*: genetics**  ***e^2^*: the individually-independent environment**  ***f^2^*: the environment shared by family members**  ***c^2^*: the environment shared by couples**  ***s^2^*: the environment shared by siblings**  ***he^2^*: the interaction between genetics and the individually-independent environment**  ***hf^2^*: the interaction between genetics and the family-shared environment**  ***hc^2^*: the interaction between genetics and the couples-shared environment**  ***hs^2^*: the interaction between genetics and the siblings-shared environment**  **WAIC**  **The widely-applicable information criterion rewards goodness of fit but penalizes more complex models. The lower the WAIC, the better the model.**  **For each disease, the WAIC-best model is annotated in red color.** | | | | | | | | | | | |

**References**

1 Bradbury, T. N. & Miller, G. A. Season of birth in schizophrenia: a review of evidence, methodology, and etiology. *Psychol Bull* **98**, 569-594, (1985).

2 Torrey, E. F., Miller, J., Rawlings, R. & Yolken, R. H. Seasonality of births in schizophrenia and bipolar disorder: A review of the literature. *Schizophr Res* **24**, 260-260, (1997).

3 Susser, E., Hoek, H. W. & Brown, A. Neurodevelopmental disorders after prenatal famine: The story of the Dutch Famine Study. *Am J Epidemiol* **147**, 213-216, (1998).

4 Hollister, J. M., Laing, P. & Mednick, S. A. Rhesus incompatibility as a risk factor for schizophrenia in male adults. *Arch Gen Psychiat* **53**, 19-24, (1996).

5 Cannon, T. D. On the nature and mechanisms of obstetric influences in schizophrenia: a review and synthesis of epidemiologic studies. *Int Rev Psychiatr* **9**, 387-397, (1997).

6 Cannon, T. D. *et al.* A prospective cohort study of genetic and perinatal influences in the etiology of schizophrenia. *Schizophr Bull* **26**, 351-366, (2000).

7 Buoli, M. *et al.* Are obstetrical complications really involved in the etiology and course of schizophrenia and mood disorders? *Psychiatry Res* **241**, 297-301, (2016).

8 Dalman, C. G., Allebeck, P., Grunewald, C. & Haglund, B. Obstetric complications and the risk of schizophrenia - A longitudinal study of a national birth cohort. *Schizophr Res* **49**, 29-29, (2001).

9 Dalman, C., Allebeck, P., Cullberg, J., Grunewald, C. & Koster, M. Obstetric complications and the risk of schizophrenia - A longitudinal study of a national birth cohort. *Arch Gen Psychiat* **56**, 234-240, (1999).

10 Fair, D. The Influence of Maternal Prenatal Stress Trajectories During Pregnancy on Offspring Brain and Behaviors From 0-24 Months of Age. *Neuropsychopharmacology* **43**, S49-S49, (2018).

11 Alonso, S. J., Arevalo, R., Afonso, D. & Rodriguez, M. Effects of Maternal Stress during Pregnancy on Forced Swimming Test Behavior of the Offspring. *Physiology & Behavior* **50**, 511-517, (1991).

12 Selten, J. P., Cantor-Graae, E., Levav, I., Nahon, D. & Kahn, R. S. Prenatal exposure to maternal stress and subsequent risk of schizophrenia: No effect of six-day war and Yom Kippur War. *Schizophr Res* **53**, 237-237, (2002).

13 Quenstedt, M. & Parshall, A. Prenatal exposure to maternal stress and subsequent schizophrenia. *Brit J Psychiat* **173**, 183-183, (1998).

14 van Os, J. & Selten, J. P. Prenatal exposure to maternal stress and subsequent schizophrenia - The May 1940 invasion of The Netherlands. *Brit J Psychiat* **172**, 324-326, (1998).

15 Smythe, J. W., Mccormick, C. M., Rochford, J. & Meaney, M. J. The Interaction between Prenatal Stress and Neonatal Handling on Nociceptive Response Latencies in Male and Female Rats. *Physiology & Behavior* **55**, 971-974, (1994).

16 Barr, C. E., Mednick, S. A. & Munk-Jorgensen, P. Exposure to influenza epidemics during gestation and adult schizophrenia. A 40-year study. *Arch Gen Psychiatry* **47**, 869-874, (1990).

17 Shi, L., Fatemi, S. H., Sidwell, R. W. & Patterson, P. H. Maternal influenza infection causes marked behavioral and pharmacological changes in the offspring. *J Neurosci* **23**, 297-302, (2003).

18 Brown, A. S. *et al.* A.E. Bennett Research Award. Prenatal rubella, premorbid abnormalities, and adult schizophrenia. *Biol Psychiatry* **49**, 473-486, (2001).

19 Suvisaari, J., Haukka, J., Tanskanen, A., Hovi, T. & Lonnqvist, J. Association between prenatal exposure to poliovirus infection and adult schizophrenia. *Am J Psychiat* **156**, 1100-1102, (1999).

20 Fearon, P. *et al.* Brain volumes in adult survivors of very low birth weight: A sibling-controlled study. *Pediatrics* **114**, 367-371, (2004).

21 Nosarti, C. *et al.* Adolescents who were born very preterm have decreased brain volumes. *Brain* **125**, 1616-1623, (2002).

22 Maki, P. *et al.* Schizophrenia in the offspring of antenatally depressed mothers: a 31-year follow-up of the Northern Finland 1966 Birth Cohort. *Schizophr Res* **66**, 79-81, (2004).

23 McGrath, J. J. *et al.* Vitamin D supplementation during the first year of life and risk of schizophrenia: A Finnish birth-cohort study. *Schizophr Res* **67**, 16-16, (2004).

24 Mulvany, F. *et al.* Effect of social class at birth on risk and presentation of schizophrenia: case-control study. *Bmj-Brit Med J* **323**, 1398-1401, (2001).

25 Khan, A. *et al.* Environmental pollution is associated with increased risk of psychiatric disorders in the US and Denmark. *PLoS Biol* **17**, e3000353, (2019).

26 Debnath, M. Adaptive Immunity in Schizophrenia: Functional Implications of T Cells in the Etiology, Course and Treatment. *Journal of neuroimmune pharmacology : the official journal of the Society on NeuroImmune Pharmacology* **10**, 610-619, (2015).

27 Anderson, G. Neuronal-immune interactions in mediating stress effects in the etiology and course of schizophrenia: role of the amygdala in developmental co-ordination. *Med Hypotheses* **76**, 54-60, (2011).

28 Gattaz, W. F., Abrahao, A. L. & Foccacia, R. Childhood meningitis, brain maturation and the risk of psychosis. *Eur Arch Psychiatry Clin Neurosci* **254**, 23-26, (2004).

29 Torrey, E. F. & Yolken, R. H. Toxoplasma gondii and schizophrenia. *Emerg Infect Dis* **9**, 1375-1380, (2003).

30 Mullen, P. E., Martin, J. L., Anderson, J. C., Romans, S. E. & Herbison, G. P. Childhood Sexual Abuse and Mental-Health in Adult Life. *Brit J Psychiat* **163**, 721-732, (1993).

31 Anderson, G. *et al.* Immuno-inflammatory, oxidative and nitrosative stress, and neuroprogressive pathways in the etiology, course and treatment of schizophrenia. *Prog Neuropsychopharmacol Biol Psychiatry* **42**, 1-4, (2013).

32 Done, D. J., Crow, T. J., Johnstone, E. C. & Sacker, A. Childhood Antecedents of Schizophrenia and Affective-Illness - Social-Adjustment at Ages 7 and 11. *Brit Med J* **309**, 699-703, (1994).

33 Norman, R. M. & Malla, A. K. Stressful life events and schizophrenia. I: A review of the research. *Br J Psychiatry* **162**, 161-166, (1993).

34 Rabkin, J. G. Stressful life events and schizophrenia: a review of the research literature. *Psychol Bull* **87**, 408-425, (1980).

35 Huttunen, M. O. & Niskanen, P. Prenatal Loss of Father and Psychiatric-Disorders. *Arch Gen Psychiat* **35**, 429-431, (1978).

36 Walker, E. F., Cudeck, R., Mednick, S. A. & Schulsinger, F. Effects of Parental Absence and Institutionalization on the Development of Clinical Symptoms in High-Risk Children. *Acta Psychiat Scand* **63**, 95-109, (1981).

37 Walker, E., Downey, G. & Bergman, A. The Effects of Parental Psychopathology and Maltreatment on Child-Behavior - a Test of the Diathesis-Stress Model. *Child development* **60**, 15-24, (1989).

38 Arseneault, L. *et al.* Cannabis use in adolescence and risk for adult psychosis: longitudinal prospective study. *Brit Med J* **325**, 1212-1213, (2002).

39 Muntjewerff, J. W. *et al.* Homocysteine metabolism and B-vitamins in schizophrenic patients: low plasma folate as a possible independent risk factor for schizophrenia. *Psychiat Res* **121**, 1-9, (2003).

40 Sachdev, P., Smith, J. S. & Cathcart, S. Schizophrenia-like psychosis following traumatic brain injury: a chart-based descriptive and case-control study. *Psychological Medicine* **31**, 231-239, (2001).

41 Mall, S. Does substance abuse mediate or moderate the relationship between childhood trauma and the experience of persecutory delusions in people with schizophrenia in South Africa? *Eur Psychiat* **41**, S246-S246, (2017).

42 Kiev, A. Psychiatric Morbidity of West-Indian Immigrants in an Urban Group-Practice. *Brit J Psychiat* **111**, 51-56, (1965).

43 Boydell, J. *et al.* Incidence of schizophrenia in ethnic minorities in London: ecological study into interactions with environment. *Bmj-Brit Med J* **323**, 1336-1338, (2001).

44 Kinney, D. K. *et al.* Relation of schizophrenia prevalence to latitude, climate, fish consumption, infant mortality, and skin color: a role for prenatal vitamin d deficiency and infections? *Schizophr Bull* **35**, 582-595, (2009).

45 Carvalho, C. M., Polson, N. G. & Scott, J. G. in *Artificial Intelligence and Statistics.* 73-80.

46 Chung, Y., Rabe-Hesketh, S., Dorie, V., Gelman, A. & Liu, J. A nondegenerate penalized likelihood estimator for variance parameters in multilevel models. *Psychometrika* **78**, 685-709, (2013).

47 Gelman, A. Prior choice recommendations. *Retrieved July* **24**, 2019, (2019).

48 Hoffman, M. D. & Gelman, A. The No-U-Turn sampler: adaptively setting path lengths in Hamiltonian Monte Carlo. *Journal of Machine Learning Research* **15**, 1593-1623, (2014).

49 Kucukelbir, A., Tran, D., Ranganath, R., Gelman, A. & Blei, D. M. Automatic differentiation variational inference. *The Journal of Machine Learning Research* **18**, 430-474, (2017).

50 Starke, L. & Ostwald, D. Variational Bayesian parameter estimation techniques for the general linear model. *Frontiers in neuroscience* **11**, 504, (2017).
